# A clinical guideline-based management of type 2 diabetes by ayurvedic practitioners in Nepal: A feasibility cluster randomized controlled trial protocol

**DOI:** 10.1097/MD.0000000000031452

**Published:** 2022-11-25

**Authors:** Kaushik Chattopadhyay, Meghnath Dhimal, Shristi Karki, Prerok Regmi, Bihungum Bista, Tuhin Kanti Biswas, Michael Heinrich, Jeemon Panniyammakal, Nikhil Tandon, Jo Leonardi-Bee, Sanjay Kinra, Sheila Margaret Greenfield, Sarah Anne Lewis, Vasudev Upadhyay, Pradip Gyanwali

**Affiliations:** a Lifespan and Population Health Academic Unit, School of Medicine, University of Nottingham, Nottingham, UK; b The Nottingham Centre for Evidence-Based Healthcare: A JBI Centre of Excellence, Nottingham, UK; c Nepal Health Research Council, Kathmandu, Nepal; d Department of Kayachikitsa, J B Roy State Ayurvedic Medical College and Hospital, Kolkata, India; e Centre for Pharmacognosy and Phytotherapy, School of Pharmacy, University College London, London, UK; f Sree Chitra Tirunal Institute for Medical Sciences and Technology, Thiruvananthapuram, India; g Department of Endocrinology, Metabolism and Diabetes, All India Institute of Medical Sciences, New Delhi, India; h Department of Non-communicable Disease Epidemiology, London School of Hygiene and Tropical Medicine, London, UK; i Institute of Applied Health Research, University of Birmingham, Birmingham, UK; j Department of Ayurveda and Alternative Medicine, Ministry of Health and Population, Kathmandu, Nepal.

**Keywords:** ayurveda, clinical guideline, feasibility trial, management, Nepal, type 2 diabetes mellitus

## Abstract

Type 2 diabetes mellitus (T2DM) is a common chronic condition with significant health and socioeconomic consequences. In Nepal, T2DM is a common disease for which people consult ayurvedic (traditional medical system) practitioners and use ayurvedic medicines. Strong concerns remain about the suboptimal T2DM management of many patients by ayurvedic practitioners, and therefore, based on the best available scientific evidence, we have developed a clinical guideline for managing T2DM by ayurvedic practitioners. The research question to be addressed by a definitive cluster randomized controlled trial (RCT) is whether the introduction of a clinical guideline can improve the management of T2DM by ayurvedic practitioners in Nepal as compared to usual ayurvedic management (i.e., without any clinical guideline). In preparation for this future work, this current study aims to determine the feasibility of undertaking the definitive cluster RCT.

**Methods:**

This is a 2-arm, feasibility cluster RCT with a blinded outcome assessment and a qualitative evaluation. The study is conducted in 12 public and private ayurveda centers in and outside the Kathmandu Valley in Nepal (1:1 intervention:control). Eligible participants should be new T2DM adult patients (i.e., treatment naïve) - the glycated hemoglobin level should be 6.5% or above but less than 9%. At least 120 participants (60/group) will be recruited and followed up for 6 months. Important parameters, needed to design the definitive trial, will be estimated, such as the standard deviation of the outcome measure (i.e., glycated hemoglobin level at 6-month follow-up), intraclass correlation coefficient, cluster size, recruitment, the time needed to recruit participants, follow-up, and adherence to the recommended ayurvedic medicine. Semi-structured qualitative interviews will be conducted with around 20 to 30 participants and all the participating ayurvedic practitioners to explore their experiences and perspectives of taking part in the study and of the intervention and a sample of eligible people declining to participate in the study to explore the reasons behind nonparticipation.

**Discussion:**

We are now conducting a feasibility cluster RCT in Nepal to determine the feasibility of undertaking the definitive cluster trial. The first participant was recruited on 17 July 2022. If the feasibility is promising (such as recruitment, follow-up, and adherence to the recommended ayurvedic medicine), then the parameters estimated will be used to design the definitive cluster trial. Decisions over whether to modify the protocol will mainly be informed by the qualitative data.

## 1. Introduction

Diabetes mellitus is one of the most common chronic diseases, and its global prevalence is increasing.^[1]^ Currently, 1 in 10 adults is living with diabetes, and around 44% are undiagnosed.^[1]^ About 90% of adults currently diagnosed have type 2 diabetes its global prevalence is increasing.[1] Currently, 1 in 10 adults is currently undiagnosed.^[1]^ T2DM is a complex metabolic disorder with significant health and socioeconomic consequences.^[1,2]^ Chronic hyperglycemia in T2DM is associated with macrovascular and microvascular complications and even death.^[1,2]^ In Nepal, the prevalence of T2DM between 2010 and 2015 was around 8% (95% confidence interval (CI): 4–16), which increased to 11% (8–16) between 2015 and 2020.^[3]^

In Nepal, ayurveda is a dominant traditional medical system and has been in use for thousands of years.^[4]^ Qualified and registered ayurvedic practitioners are employed in the public and private healthcare system (e.g., in ayurveda centers), often as the main clinical provider.^[5]^ The corresponding term for diabetes mellitus in ayurveda is madhumeha (madhu means “sweetness” and meha means “excessive urination”).^[6,7]^ The condition and its management have been described in detail in classical ayurvedic texts.^[6,7]^ Briefly, a multipronged approach is used to manage the condition, such as through lifestyle changes and ayurvedic medicines (containing plant-, animal-, or mineral-origin ingredients–single or in combination). It is hypothesized that many of these ayurvedic medicines work through pancreatic and extrapancreatic effects.^[6,7]^ T2DM is one of the common diseases for which patients consult ayurvedic practitioners, and many people with T2DM use ayurvedic medicines, often continuously from the point of diagnosis.^[5,8]^ Ayurveda fits their health beliefs and culture, and acceptability, satisfaction, and perceived relief are usually high, especially among rural, poor, older, and tribal populations.^[8–10]^ Many people with T2DM prefer not to use western medicines in order to avoid their side effects, cost, and mode of administration like insulin injections.^[8,9,11,12]^

Strong concerns remain about the suboptimal T2DM management of many patients by ayurvedic practitioners, and the actions to be taken at different stages of the T2DM care pathway are largely left to the judgment of the individual ayurvedic practitioner (including screening for complications and referral to specialist services for complications management), resulting in unacceptable variations in ayurvedic clinical practice.^[13–15]^ Many nonevidence-based ayurvedic medicines are prescribed by them, which can have serious adverse effects on patients, including heavy metal poisoning.^[16]^ Many times ayurvedic practitioners blindly follow the claims made by others or opt for a “trial and error” approach.^[15,17]^ One of the main barriers highlighted by ayurvedic practitioners is the absence of a good quality clinical guideline to deliver quality care to people with T2DM.^[15]^

In western medicine, clinical guidelines have been used to improve the clinical care of people with T2DM.^[18,19]^ However, no such clinical guideline for managing T2DM is available in Nepal for ayurvedic practitioners. A good quality clinical guideline, based on the best available scientific evidence, to manage T2DM by ayurvedic practitioners may address the existing problems and deter the usage of ayurvedic medicines of no, minimal, or questionable value and promote the usage of effective and safe ayurvedic medicines. It will aid their clinical decision-making process through recommended actions at various stages of the T2DM care pathway. It may close the gap between what they do to manage T2DM and what the scientific evidence supports.

The introduction of a clinical guideline is not always effective in improving the outcomes (including patients’ health), and it needs to be evaluated in the target population and healthcare setting before scaling up.^[20–22]^ This is particularly important in this case where an innovative approach is used to integrate ayurvedic and western medical systems. Therefore, the principal research question to be addressed by a definitive cluster randomized controlled trial (RCT) is whether the introduction of a clinical guideline can improve the management of T2DM by ayurvedic practitioners in Nepal as compared to usual ayurvedic management. In preparation for this future work, this current study aims to determine the feasibility of undertaking the definitive cluster RCT. The chances of successful completion of a costly definitive trial improve if the feasibility of its key elements is checked before it starts, and important parameters, needed to design the definitive trial, are estimated.^[23]^

## 2. Methods

### 2.1. Study design

This is a 2-arm, feasibility cluster RCT with a blinded outcome assessment and a qualitative evaluation.

### 2.2. Study setting

The study is conducted in 12 public and private ayurveda centers (clusters) in and outside the Kathmandu Valley in Nepal. These centers have a minimum of one ayurvedic practitioner with at least 5½ years of an undergraduate medical degree in ayurveda. All the centers approached are participating in the study. People from a wide range of socioeconomic backgrounds access these centers.

### 2.3. Randomization and blinding

Ayurveda centers are randomized to the intervention or control group according to a computer-generated randomization schedule (1:1 using Random Allocation Software Version 1.0), performed by an independent statistician at the Nepal Health Research Council, Kathmandu, Nepal. Ayurvedic practitioners and participants cannot be “blinded” to group allocation, but the outcome assessors are “blind”. The outcome assessors are an independent team of trained researchers. To give some protection against contamination, intervention group ayurvedic practitioners have signed a nondisclosure form, and they are not allowed to disclose the content of the intervention to anyone.

### 2.4. Intervention

In the intervention group, ayurvedic practitioners will manage T2DM based on a clinical guideline developed by us. The intervention development process will be published elsewhere, and the clinical guideline is based on the best available scientific evidence. Briefly, a systematic process was followed to develop the clinical guideline, guided by the Grading of Recommendations, Assessment, Development and Evaluation (GRADE) approach, the UK’s National Institute for Health and Care Excellence manual for developing guidelines, and the Appraisal of Guidelines for Research and Evaluation (AGREE) II instrument.^[24–26]^ Initially, we conducted a comprehensive systematic review assessing the effectiveness and safety of ayurvedic medicines for managing T2DM and the certainty of the findings using the GRADE approach.^[27]^ Subsequently, based on the GRADE approach, we developed the Evidence-to-Decision framework, focusing on glycemic control and adverse events. Based on the Evidence-to-Decision framework, a Guideline Development Group, comprising 17 international members, made recommendations on the effectiveness and safety of ayurvedic medicines in T2DM. The recommendations formed the basis of a clinical guideline, with further generic content and recommendations adapted from the T2DM Clinical Knowledge Summaries of the Clarity Informatics (UK).^[28]^ The Guideline Development Group gave feedback on the draft version, which was used to amend and finalize the clinical guideline. The clinical guideline will support ayurvedic practitioners in providing appropriate care, education, and support to participants (and their carers and family). The clinical guideline covers topics like diagnosis, management through lifestyle advice and ayurvedic medicines, screening for complications, and referral to specialist services for complications management. Regular training is provided to ayurvedic practitioners in the use of the clinical guideline which involves roleplaying and structured and instructive feedback to improve their performance. To maximize the implementation of the clinical guideline, a prescription pad based on the clinical guideline is provided to the ayurvedic practitioners to help them to provide relevant advice to the participants, and an ayurvedic medicine recommended in the clinical guideline is provided free of cost to the participants for the duration of the study.

### 2.5. Control

No clinical guideline is available in Nepal to manage T2DM by ayurvedic practitioners. Thus, the comparator will be the usual management of T2DM (i.e., without any clinical guideline) by ayurvedic practitioners.

### 2.6. Eligibility criteria

#### 2.6.1. Inclusion criteria

Participants should be:

- Adults (aged ≥ 18 years).
- New T2DM patients (i.e., treatment naïve to any blood glucose-lowering medication) - the glycated hemoglobin (HbA1c) level should be 6.5% or above^[28]^ but less than 9%.

#### 2.6.2. Exclusion criteria

- Pregnant women.
- Those with any serious or uncontrolled medical condition (e.g., cancer).
- Those currently receiving (or with plans to receive during the study period) any related non-pharmaceutical or pharmaceutical research intervention.

### 2.7. Screening, recruitment, and follow-up

Adults visiting the ayurveda centers with possible clinical features of T2DM and/or hyperglycemia (i.e., HbA1c of 6.5–9%, fasting blood glucose of ≥ 126 mg/dL, or random blood glucose ≥ 200 mg/dL) but treatment naïve are approached for screening. The participant information sheet is given to these potential participants, including a verbal description of the study and answering their questions. Those interested in the study are requested to provide written informed consent. The participant information sheet and consent form are available in Nepali and English. Those providing written informed consent are assessed against the study eligibility criteria, and their HbA1c is determined using a venous blood sample (see Table 1). After the recruitment of a participant, baseline data are collected by “blind” outcome assessors. Each participant will be followed up for 6 months.

**Table 1:** Data collection.

		Face-to-face assessments		
	Assessment details	Screening and recruitment	Baseline	Final at 6-mo follow-up (±5 d)
Eligibility assessment		√		
Sociodemographics	Patient reported		√	
Medical and surgical history	Patient reported		√	
Family history of diabetes	Patient reported		√	
Current concomitant medications (any medical system)	Patient reported		√	√
Biochemical parameters				
*Blood glucose*				
Glycated hemoglobin*	High-performance liquid chromatography (HPLC) method	√		√
Fasting blood glucose†	Glucose oxidase-peroxidase (GOD-POD) method		√	√
*Lipid profile*				
Total cholesterol	Cholesterol oxidase method		√	√
High-density lipoprotein	Direct clearance method		√	√
Low-density lipoprotein	Direct clearance method		√	√
Very low-density lipoprotein	Calculated value		√	√
Triglyceride	Lipase/glycerol-3-phosphate oxidase-phenol + aminophenazone (GPO-PAP) no correction method		√	√
*Liver function tests*				
Albumin	Spectrophotometry (Bromocresol green method)		√	√
Total bilirubin	Spectrophotometry (modified Jendrassik and Grof method)		√	√
Direct bilirubin	Spectrophotometry (modified Jendrassik and Grof method)		√	√
Indirect bilirubin	Spectrophotometry (modified Jendrassik and Grof method)		√	√
Alkaline phosphatase	Ultraviolet (UV) kinetic (modified IFCC method)		√	√
Alanine transaminase (ALT)	UV kinetic (modified IFCC method)		√	√
Aspartate transaminase (AST)	UV kinetic (modified International Federation of Clinical Chemistry and Laboratory Medicine (IFCC) method)		√	√
AST-ALT ratio	Calculated value		√	√
*Electrolytes*				
Sodium	Ion selective electrode (ISE)		√	√
Potassium	ISE		√	√
Urea	Spectrophotometry (Berthelot method)		√	√
Creatinine	Spectrophotometry (modified Jaffe’s method)		√	√
*Urine albumin-to-creatinine ratio*	Spectrophotometry		√	√
Physiological parameters†				
Blood pressure	Omron HEM-8712		√	√
Heart rate	Omron HEM-8712		√	√
Anthropometric parameters†				
Waist circumference	Seca 201 (measuring tape)		√	√
Bodyweight	Dr Care (weighing scale)		√	√
Height	Stature meter		√	√
Body mass index	Calculated value		√	√
Lifestyle				
Diet	Patient reported		√	√
Physical activity	Patient reported International Physical Activity Questionnaire (IPAQ)–Short^[29]^		√	√
Tobacco usage	Patient reported		√	√
Alcohol consumption	Patient reported		√	√
Health-related quality-of-life	Patient reported EuroQol-5D-5L (EQ-5D-5L)^[30]^		√	√
Depression, anxiety, and stress	Patient reported Depression, Anxiety and Stress Scale (DASS-21)^[31]^		√	√
Perception of illness	Patient reported Brief Illness Perception Questionnaire (BIPQ)^[32]^		√	√
Satisfaction with treatment	Patient reported Short Assessment of Patient Satisfaction (SAPS)^[33]^			√

* Also assessed at 3-month follow-up.

† Also assessed at 1-month follow-up.

### 2.8. Study parameters and data collection

#### 2.8.1. Cluster RCT

- For sample size calculation of the definitive cluster RCT:
  - The standard deviation (SD) of the primary outcome measure (i.e., HbA1c level at 6-month follow-up).
  - Another estimate of intraclass correlation coefficient and cluster size (i.e., the number of participants recruited per ayurveda center during the recruitment period).
- Recruitment: The number of people approached to participate, written informed consent given, screened for eligibility, found eligible, and recruited.
- The time needed to recruit participants.
- Follow-up: The number of participants followed up for 6 months.
- Adherence: In the intervention group, the number of participants adhering to the recommended ayurvedic medicine (assessed using a self-reported diary and by medicine counting at the sites).
- Others: See Table 1.

A standard operating procedure is developed and used for data collection. Blood and urine samples are collected and analyzed at local laboratories, accredited by the National Public Health Laboratory in Nepal.

### 2.8.2. Qualitative evaluation

- Interviews will be conducted with participants to explore their experiences and perspectives of taking part in the study (intervention and control group participants who complete or do not complete the study) and of the intervention (intervention group only).
- Eligible people who decline to participate in the study will be requested to complete a questionnaire (including reasons behind nonparticipation). A sample of these people (who agree to be interviewed) will be interviewed to further explore their reasons for nonparticipation.
- Interviews will be conducted with all the participating ayurvedic practitioners to explore their experiences and perspectives of taking part in the study (both the groups) and of the intervention (intervention group only and focusing on topics like its acceptability and factors that facilitated or impeded its uptake and adherence).

Predeveloped interview guides will be used by a qualitative researcher to conduct these semi-structured interviews. The interviews will be conducted in the interviewees’ preferred language and with the help of an interpreter if needed. With consent, these will be noted and digitally recorded.

### 2.9. Data analyses

#### 2.9.1. Cluster RCT.

We will summarize the data using numbers and percentages for categorical data and summary measures of mean or median and spread for continuous data. Being a feasibility cluster RCT, it is not adequately powered to detect a difference in trial outcomes between the 2 arms. However, unadjusted mean difference or odds ratio with 95% CI will be reported to indicate initial estimates of effects. Subsequently, for critical outcomes like HbA1c, ANCOVA analysis will be conducted, allowing for clustering and adjustments made for the individual’s baseline value and subsequently, also for the individual’s age and sex and cluster (ayurveda center) mean.^[34]^ The regression coefficient and 95% CI will be reported. All analyses will be based on the intention-to-treat principle. Being a short-term feasibility study, no interim analysis is planned. STATA V.17 will be used for data analyses.

#### 2.9.2. Qualitative evaluation.

All the semi-structured interviews will be transcribed (verbatim), translated to English (if necessary), anonymized, and checked for accuracy. For transcription and translation, encrypted files will be transferred to an external transcription and translation company after signing the non-disclosure agreement. An interpretive analysis will be conducted using thematic analysis,^[35]^ using NVivo software (2020). Transcripts will be read and re-read. Initial codes will be developed and applied initially to a small number of transcripts, enabling further iteration of the thematic index which will then be applied and refined across all the transcripts. We will use illustrative non-attributable quotations.

### 2.10. Sample size

#### 2.10.1. Cluster RCT.

A formal sample size calculation is not usually required for a feasibility trial, and it is recommended to recruit at least 50 participants in a feasibility trial.^[36]^ After taking into account the cluster study design and loss to follow-up, we will recruit at least 120 participants (60/group) in this feasibility cluster RCT to estimate the SD of the primary outcome measure (i.e., HbA1c level at 6-month follow-up). This sample size is calculated in relation to the desired level of confidence (95%) for the SD, the chosen power (80%) and significance level (5%, 2-tailed), and the expected loss to follow-up (20% at 6 months), intraclass correlation coefficient (0.03), and mean cluster size (10 participants).^[36–38]^

#### 2.10.2. Qualitative evaluation

- Interviews will be conducted with around 20 to 30 participants. Until data saturation is achieved, purposive sampling will be used for ensuring the representation of diversity within the cluster RCT population.^[35]^
- Interviews will be conducted with around 10 to 15 people (continue until data saturation is reached) who decline to participate in the study but agree to be interviewed about their reasons for nonparticipation.^[35]^
- Interviews will be conducted with all the participating ayurvedic practitioners (continue until data saturation is reached).^[35]^

### 2.11. Ethics and other related issues

Ethics approval has been obtained from the following: Research Ethics Committee, Faculty of Medicine and Health Sciences, University of Nottingham, UK (511-2003) and Ethical Review Board, Nepal Health Research Council, Nepal (66/2022). The clinical trial license has been received from the Department of Drug Administration, Nepal. An independent Trial Steering Committee, consisting of relevant experts, is monitoring and providing overall supervision for the study.

### 2.12. Adverse events and serious adverse events

Information will be collected on adverse events and serious adverse events (including death) occurring in participants. Based on medical and scientific judgment, an independent clinician (diabetologist) will determine the relationship of any serious adverse event to the interventions.

### 2.13. Participant withdrawal

Participants will be withdrawn from the study either at their request or at the discretion of the site investigator (e.g., if a female participant gets pregnant during the study period, if a participant develops a serious or uncontrolled medical condition (e.g., cancer) during the study period). Participants will be made aware that this will not affect their future care. Also, they will be made aware (via the participant information sheet and consent form) that should they withdraw, the data collected to date will not be erased and may still be used in the final analyses.

### 2.14. Dissemination

The findings will be reported according to the relevant extension of the Consolidated Standards of Reporting Trials Statement (i.e., for randomized pilot and feasibility trials).^[39]^ The findings will be widely disseminated among key stakeholders through various avenues, such as through dissemination meetings and informal discussions with them, presentations at national and international conferences, publications in peer-reviewed open-access journals, and press offices and websites of host institutions.

## 3. Discussion

We are now conducting a feasibility cluster RCT in Nepal to determine the feasibility of undertaking the definitive cluster trial. The first participant was recruited on 17 July 2022. If the feasibility is promising (such as recruitment, follow-up, and adherence to the recommended ayurvedic medicine), then the parameters estimated will be used to design the definitive cluster trial. Decisions over whether to modify the protocol will mainly be informed by the qualitative data. We intend to do long-term (≥1 yr) follow-ups in the definitive cluster RCT.

If the intervention is found to be effective in the definitive cluster RCT, people with T2DM in Nepal will benefit from improved health outcomes, such as better blood glucose control and lower T2DM complications. The related clinical, personal, and economic burden on people with T2DM and their carers and family will be reduced. People with T2DM will be cared for in line with the best available scientific evidence and in the same manner regardless of where or by which ayurvedic practitioner they are treated. Given that T2DM is a global concern, the clinical guideline will be of interest in other countries, particularly in neighboring South Asian nations and in countries with South Asian ethnic minorities who often rely on ayurvedic treatments.^[40,41]^

Health policymakers and managers will benefit from the availability of an effective solution to manage T2DM by ayurvedic practitioners. The related economic burden on the health system and economy will be reduced. The clinical guideline may improve the efficiency of healthcare through standardized care, providing better value for money. In the case of ayurvedic medicines of no, minimal, or questionable value, they will benefit from the identified disinvestment opportunities, cost savings, and opportunities for redirecting the resources (from ineffective to effective ayurvedic medicines). The provision of evidence-based healthcare and reduction in unacceptable variations in ayurvedic clinical practice will send messages of commitment to excellence and quality, thus building people’s confidence in the health system and improving the public image.

## Acknowledgments

The authors would like to thank the funding agencies, study participants, and Trial Steering Committee members, namely, Prof Prakash Ghimire (Chair), Dr Kiran Acharya, Dr Archana Shrestha, Dr Rajendra Gyanwali, and Prof Nitin Kapoor.

## Author contributions

KC conceptualized and designed the study with the help of MD, BB, TKB, JP, NT, SK, MH, JLB, SMG, SAL, VU, and PG. KC wrote the first draft of the manuscript. MD, SK, PR, BB, TKB, JP, NT, SK, MH, JLB, SMG, SAL, VU, and PG contributed significantly to the revision of the manuscript. All authors read and approved the final manuscript.

**Conceptualization:** Kaushik Chattopadhyay, Meghnath Dhimal, Bihungum Bista, Tuhin Kanti Biswas, Michael Heinrich, Jeemon Panniyammakal, Nikhil Tandon, Jo Leonardi-Bee, Sanjay Kinra, Sheila Margaret Greenfield, Sarah Anne Lewis, Pradip Gyanwali.

**Funding acquisition:** Kaushik Chattopadhyay, Tuhin Kanti Biswas, Michael Heinrich, Jeemon Panniyammakal, Nikhil Tandon, Jo Leonardi-Bee, Sanjay Kinra, Sheila Margaret Greenfield, Sarah Anne Lewis.

**Investigation:** Kaushik Chattopadhyay, Meghnath Dhimal, Shristi Karki, Prerok Regmi, Bihungum Bista, Tuhin Kanti Biswas, Michael Heinrich, Jeemon Panniyammakal, Nikhil Tandon, Jo Leonardi-Bee, Sanjay Kinra, Sarah Anne Lewis, Vasudev Upadhyay, Pradip Gyanwali.

**Methodology:** Kaushik Chattopadhyay, Meghnath Dhimal, Shristi Karki, Prerok Regmi, Bihungum Bista, Tuhin Kanti Biswas, Michael Heinrich, Jeemon Panniyammakal, Nikhil Tandon, Jo Leonardi-Bee, Sanjay Kinra, Sheila Margaret Greenfield, Sarah Anne Lewis, Vasudev Upadhyay, Pradip Gyanwali.

**Project administration:** Kaushik Chattopadhyay, Meghnath Dhimal, Shristi Karki, Prerok Regmi, Bihungum Bista, Vasudev Upadhyay, Pradip Gyanwali.

**Resources:** Kaushik Chattopadhyay, Meghnath Dhimal, Shristi Karki, Prerok Regmi, Bihungum Bista, Vasudev Upadhyay, Pradip Gyanwali.

**Supervision:** Kaushik Chattopadhyay, Meghnath Dhimal, Shristi Karki, Prerok Regmi, Bihungum Bista, Vasudev Upadhyay, Pradip Gyanwali.

**Writing – original draft:** Kaushik Chattopadhyay.

**Writing – review & editing:** Kaushik Chattopadhyay, Meghnath Dhimal, Shristi Karki, Prerok Regmi, Bihungum Bista, Tuhin Kanti Biswas, Michael Heinrich, Jeemon Panniyammakal, Nikhil Tandon, Jo Leonardi-Bee, Sanjay Kinra, Sheila Margaret Greenfield, Sarah Anne Lewis, Vasudev Upadhyay, Pradip Gyanwali.
